# All-Optical Photoacoustic Sensors for Steel Rebar Corrosion Monitoring

**DOI:** 10.3390/s18051353

**Published:** 2018-04-27

**Authors:** Cong Du, Jones Owusu Twumasi, Qixiang Tang, Xu Guo, Jingcheng Zhou, Tzuyang Yu, Xingwei Wang

**Affiliations:** 1 Department of Electrical and Computer Engineering, University of Massachusetts Lowell, 1 University Ave., Lowell, MA 01854, USA; Cong_Du@student.uml.edu (C.D.); Xu_Guo@student.uml.edu (X.G.); Jingcheng_Zhou@student.uml.edu (J.Z.); 2 Department of Civil and Environmental Engineering, University of Massachusetts Lowell, 1 University Ave., Lowell, MA 01854, USA; Jones_OwusuTwumasi@student.uml.edu (J.O.T.); Qixiang_Tang@student.uml.edu (Q.T.); Tzuyang_Yu@uml.edu (T.Y.)

**Keywords:** photoacoustic principle, gold nanocomposites, FBG, distributed sensing, rebar corrosion monitoring

## Abstract

This article presents an application of an active all-optical photoacoustic sensing system with four elements for steel rebar corrosion monitoring. The sensor utilized a photoacoustic mechanism of gold nanocomposites to generate 8 MHz broadband ultrasound pulses in 0.4 mm compact space. A nanosecond 532 nm pulsed laser and 400 μm multimode fiber were employed to incite an ultrasound reaction. The fiber Bragg gratings were used as distributed ultrasound detectors. Accelerated corrosion testing was applied to four sections of a single steel rebar with four different corrosion degrees. Our results demonstrated that the mass loss of steel rebar displayed an exponential growth with ultrasound frequency shifts. The sensitivity of the sensing system was such that 0.175 MHz central frequency reduction corresponded to 0.02 g mass loss of steel rebar corrosion. It was proved that the all-optical photoacoustic sensing system can actively evaluate the corrosion of steel rebar via ultrasound spectrum. This multipoint all-optical photoacoustic method is promising for embedment into a concrete structure for distributed corrosion monitoring.

## 1. Introduction

Steel-reinforcing bars (rebars) are commonly used in reinforced and prestressed concrete (RC/PC) structures (e.g., buildings, bridges), mainly to provide tensile strength for the structures. However, corrosion of steel rebar can significantly undermine the structural integrity and reduce durability of concrete structures. Steel rebar corrosion results in a tremendous cost (more than 14 billion dollars per year in the United States) for maintenance, rehabilitation, and rebuild [1]. Furthermore, steel rebar corrosion is associated with concrete cracking and spalling, leading to loss of cross-sectional area and reduction of structural stiffness. Consequently, the toughness and ductility of RC/PC structures are jeopardized, and structures will fail in a premature, brittle mode (sudden failure). Therefore, health monitoring and damage detection of RC/PC structures for early-stage steel rebar corrosion are essential to reduce maintenance cost and to prevent sudden failure from happening [2]. Nondestructive testing (NDT) methods—such as impact-echo [3], ultrasonic testing [4], half-cell potential sensing, ground penetrating radar, and imaging radar [5]—have been used for health monitoring of concrete infrastructures. However, for inaccessible structural members, such as rebar embedded in concrete, application of the aforementioned techniques becomes very difficult.

By taking advantage of their minimum size (~0.2–0.5 mm) and strong resilience to harsh environments [6], fiber optic sensors (FOS) show a promising potential for early-stage detection of steel rebar corrosion inside RC/PC structures, especially for new construction. New generation of FOS and their packing approaches enable FOS to be integrated into concrete structures without causing significant impact to structural integrity. In long distance sensing methods, the FOS technique is mainly based on spectrum demodulation and light intensity demodulation to provide a large number of sensor nodes [7]. These sensor nodes are distributed along the full length of an optical fiber at certain intervals/distances to obtain temperature and strain information.

Most distributed fiber optic sensors are passive in the laboratory and in the practical application of civil engineering. Such sensors include grating-based sensors, Fabry-Perot interferometry-based sensors, and scattering-based distributed sensors [7,8,9]. Passive, distributed fiber optic sensors are mainly based on the principle of reflectometry, in which a light ray or wave is transmitted into the fiber and then backscattered along the fiber. The received backscattered signals are processed by a photodetector. If the fiber in the detection area is disturbed by displacement, strain, or temperature, the backscattered signals will show variation when compared to the transmitted signals. The passive method only requires a single fiber to perform large-scale monitoring. However, most applications using passive method are for one-dimensional (line) sensing [9]. If a corroded area is not covered by the optical fiber, passive sensors cannot detect the presence of corrosion, especially for early-stage corrosion [10,11].

To overcome the limitation associated with passive method/sensors, active fiber optic sensors based on photoacoustic excitation have been proposed for NDT and structure health monitoring [12,13]. This is achieved by ultrasound propagation on the target object and analysis of received signals from sensors. In active detection devices, the ultrasound generators and signal receivers are both discrete. This expands the detected area from a one-dimensional line to a two-dimensional surface between the generator and the receiver. However, current active detection methods do not possess the multiplexable feature for large-scale monitoring.

A piezoelectric transducer (PZT) is usually utilized as an ultrasound generator in NDT and FOS. However, PZT is easy to fail in a corrosion environment and to suffer electromagnetic interference. It also has highly rigorous requirements for contact area. Drawbacks include bulky size, high price, and non-integration with distributed detection, which contribute to field difficulties like the decline in testing precision dependent on the thickness of concrete.

In order to satisfy the practical monitoring requirements of remote sensing such as low cost and resistance to harsh environments, compact optical ultrasound transmitters (~0.4 mm in diameter) have recently been used to generate broad bandwidth and high-frequency ultrasound [14,15,16,17,18]. These materials are immune to electromagnetic effects due to the fact that all optical materials are as defined by the photoacoustic (PA) principle. Fiber optic photoacoustic techniques have become a promising choice for NDT in structure health monitoring [13,14]. In this study, we demonstrate the design, fabrication, and demonstration of active multipoint all-optical photoacoustic sensors. The photoacoustic generators were made of a high-efficiency nanocomposite material surrounding the optical fiber [18,19,20]. The ultrasound receivers were created from fiber Bragg grating (FBG). The ultrasound waves could be transmitted via the surface of the steel rebar to impinge on the FBG sensors. The different levels of corrosion were performed by accelerated corrosion testing. The micro-corrosion performance on the surface of the steel rebar at initial stages were evaluated by the changes in the characteristics of ultrasound waves.

This paper is organized as follows. Section 2 describes the methodology of distributed all-optical ultrasound corrosion monitoring of steel rebar and Section 3 is the experimental setup. Section 4 presents the results and discussion and Section 5 concludes the paper.

## 2. Principle of Corrosion Monitoring

### 2.1. Principle of Rebar Corrosion Monitoring

Ultrasound detection has been an effective and accurate method for structure health monitoring in civil engineering. The primary characteristics of ultrasound waves include the propagating velocity, frequency, and energy components. All these properties represent the status of the transmitting medium. Here, it was utilized to evaluate the surface performance of steel rebar. As the rebar corrosion increases, corrosion products (like pitting and rust) change the surface performance of steel rebar. These changes impact the propagation of ultrasound waves and contribute to the blocking, reflection, and absorption of ultrasound energy, which leads to an attenuation in the parameters of characteristics of ultrasound [21,22]. Based on such variation of ultrasound properties, steel rebar corrosion would respond via the frequency domain of ultrasound signals [15].

**Figure 1:**
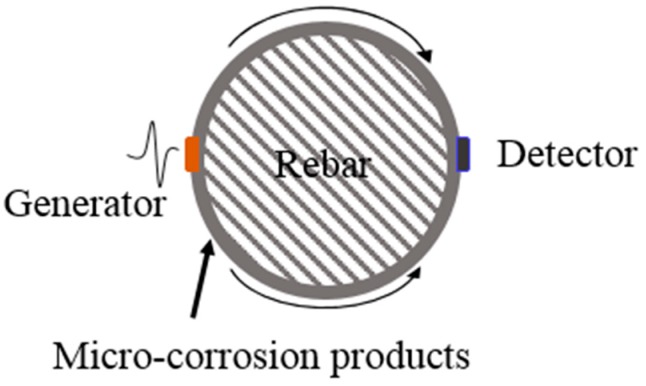
The principle of ultrasound corrosion monitoring.

As shown in Figure 1, the ultrasound generator and detector were attached on opposite sides of steel rebar. The ultrasound waves, excited by the ultrasound generator, propagated on the surface of steel rebar. Opposite of the generator, the ultrasound detector was employed to detect the signals from steel rebar. The irregularities’ microstructure changes in the steel rebar profile surface can respond once the transmission ultrasound waves impinge the ultrasound detector.

In this study, 300 mm No. 4 steel rebar with a nominal diameter of 12.7 mm was applied. To analyze the ultrasound characteristics obtained from the steel rebar, the property constants of steel rebar and ultrasound velocities in different models are shown in Table 1 [23].

### 2.2. Photoacoustic Sensors

The photoacoustic sensor was made of a composite material and an optical fiber. It was based on photoacoustic mechanism to convert light energy into the ultrasound waves. Gold nanocomposites were used as photoacoustic medium to absorb and accumulate light energy. Due to the thermal deformation effect, the switched light energy caused the expansion and contraction of the gold nanocomposites. The periodic deformation of the gold nanocomposites generated the ultrasound signals. The gold nanocomposites—which were fabricated out of polydimethylsiloxane (PDMS) mixed with gold salt (HAuCl_4_·3H_2_O)—absorbed the 532 nm laser energy, which takes high energy conversion efficiency and converts it into the ultrasound waves [20].

**Figure 2:**
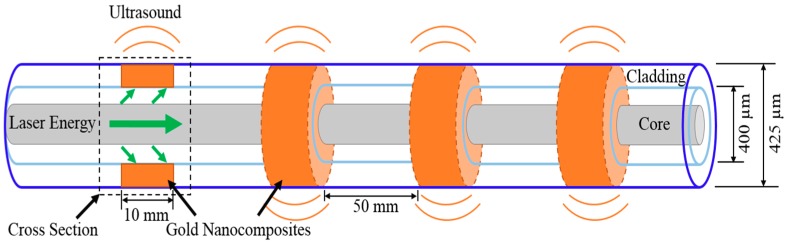
The schematic of distributed photoacoustic sensor.

The distributed photoacoustic sensor was based on evanescent wave to be fabricated on the sidewall structure of 400/425 μm core/cladding multimode fiber (MMF). As shown in Figure 2, the MMF was stripped, cladding at a length of 10 mm, to form the sidewall structures and the distance between sidewall structures was 50 mm. In this study, four sidewall structures were made in total. The structure of ultrasound generators was designed to attach to the steel rebar by coating gold nanocomposite on the core of sidewall structures.

### 2.3. Distributed Fiber Bragg Grating Sensors

The ultrasound detector is based on the variable wavelengths of FBG with detectible physical parameters such as temperature, vibration, strain, and pressure. The transmission or reflection spectrum is commonly applied to monitoring methods. In this application, a typical FBG was employed with the Bragg wavelength *λ_B_*, and is given by

**Figure 3:**
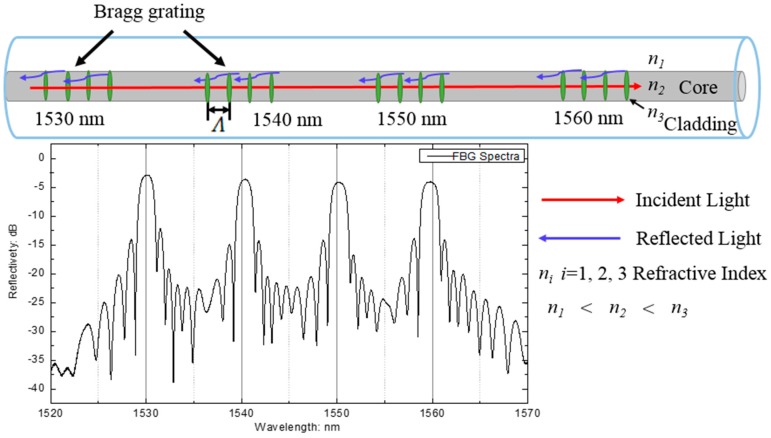
Optical reflection spectrum of the wavelength-division multiplexing techniques.

*λ_B_ = 2n_e_ Λ*, (1)

where *n_e_* and *Λ* are the effective refractive index of the fiber core and the grating period, respectively. The ultrasound detector part of our system was built based on the wavelength-division multiplexing techniques, as shown in Figure 3. The four FBGs were multiplexed in a single mode fiber. The central spectra are 1530 nm, 1540 nm, 1550 nm, and 1560 nm, respectively. Because of the continuous-wave mode of FBG sensor array, the wavelength of each sensor does not overlap and requires occupation of a distinct wavelength window [23].

## 3. Experiment Setup

### 3.1. Experimental Setup for Ultrasound Corrosion Monitoring

**Figure 4:**
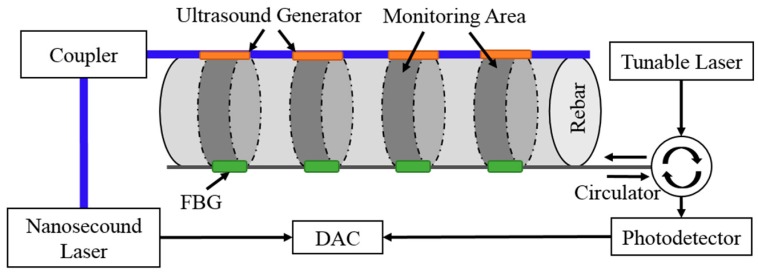
Setup of distributed ultrasound corrosion monitoring.

The schematic diagram of all-optical ultrasound corrosion sensing system is shown in Figure 4. The pulsed laser, emitted by nanosecond laser (Surelite-I-10, Continuum, San Jose, CA, USA), was coupled into MMF (UM22-400, Thorlabs, Newton, NJ, USA). The nanosecond laser source was Nd:YAG laser with 10 Hz repetition rate and 6 ns pulse-width. The laser energy was 200 mJ. The pulsed laser shot the gold nanocomposite attached on the side wall of MMF to generate the ultrasound waves. The ultrasound waves transmitted directly along the circumstantial surface of rebar to the FBG detectors.

To localize the corrosion position and degree, the FBG sensors were modulated by means of light intensity demodulation in four different wavelengths by using a tunable laser (Venturi TM Tunable Laser, TLB-6600, Santa Clara, CA, USA) as a probe light [9]. As the ultrasound vibration impinged the FBG sensors, the shifts of the central spectrum reflected to the photodetector via a circulator. The photodetector (PDA10CS, Thorlabs), with correspondence to the ultrasound signal power, could transfer the light signal to voltage magnitude. Finally, the data acquisition card (DAC) embedded in the computer recorded the voltage signals.

### 3.2. Preparation of Ultrasound Sensors on Steel Rebar

**Figure 5:**
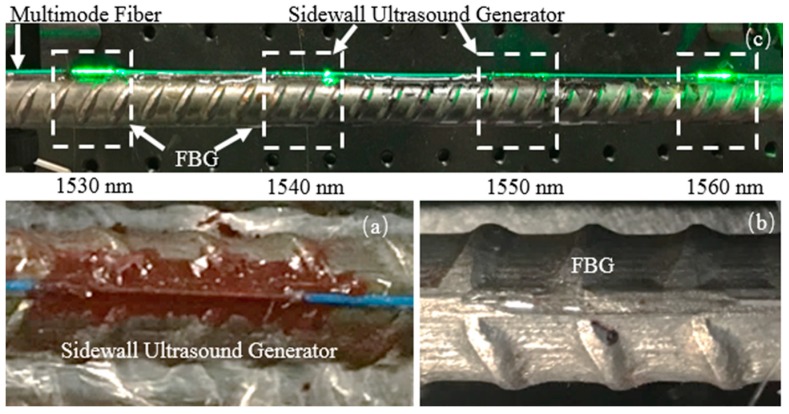
The fiber photoacoustic sensors. (**a**) The photo of the sidewall photoacoustic (PA) ultrasound generators attached on a rebar. (**b**) The photo of the fiber Bragg grating (FBG) detectors attached on a rebar. (**c**) The position of distributed photoacoustic sensors.

Before preparation for the generator and detector, the steel rebar was cleaned with acetone. There were four steps for fabrication of the distributed photoacoustic sensor on the steel rebar: (1) The four 10 mm length cladding on the MMF were removed to expose the core; (2) the MMF was bound by tape along the rib of the steel rebar, and four cores were kept in the middle position of the rib of the steel rebar; (3) gold nanocomposites were coated on the cores, attaching on the rib of the steel rebar to form the sidewall ultrasound generators; (4) the ultrasound generators were cured for 3 days on a hot plate at 120 °C. On the opposite of the generator, the epoxy (Gorilla) was then used for fixing the FBG (FBG length: 1 mm, CW: 1550 ± 0.5 nm, FWHM: 1.5 ± 0.2 nm) detector along the rib of rebar [6]. Finally, an ultraviolet glue (365A, Agiltron, Woburn, MA, USA) was covered on the fibers for protection. The entire all-optical photoacoustic sensors are shown in Figure 5c.

### 3.3. Preparation of Steel Rebar Corrosion Monitoring

**Figure 6:**
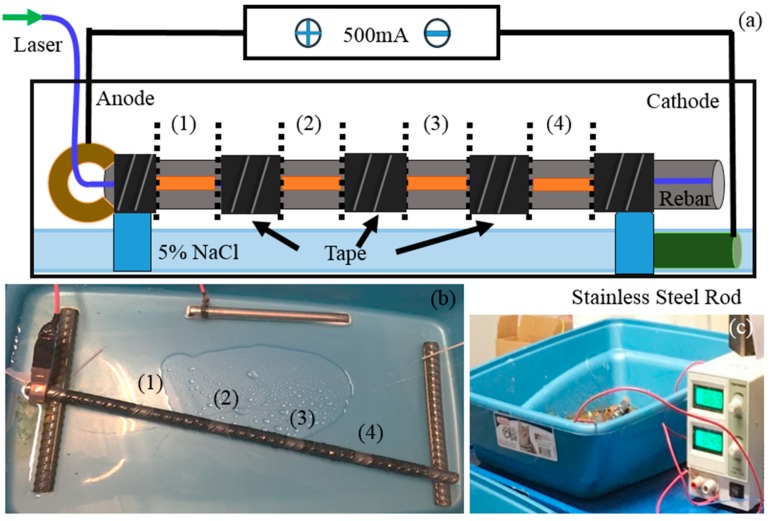
The experimental setup of corrosion monitoring on steel rebar specimen. (**a**) Schematic of accelerated corrosion test (ACT) setup. (**b**) Four spraying sections of steel rebar specimen. (**c**) Keeping a constant current of 500 mA.

To monitor different levels of corrosion on a single steel rebar using a sensing system, an accelerated corrosion test (ACT) setup was applied in Figure 6. The No. 4 steel rebar was covered with electrical insulation tape leaving only four 10 mm sections and the two ends exposed. The specimen was then suspended in a plastic container and connected to a power supply, which impressed a constant current of 500 mA. A 5% (by mass) sodium chloride (NaCl) solution was sprayed onto the specimens at an interval of 24 h. At each 24 h interval, the specimen was rotated at an angle of 180° to ensure uniform corrosion level. The steel rebar served as the anode and a 12.7 mm diameter stainless-steel rod served as the cathode.

To achieve four different corrosion levels on the single steel rebar, the four exposed sections marked (1), (2), (3), and (4), excluding the two ends on the steel rebar, were sprayed with 5% NaCl solution at different frequencies, shown in Table 2. The accelerated corrosion scheme was designed to ensure that rust products were bonded to the surface of the steel rebar at the end of the corrosion process.

## 4. Results and Discussion

### 4.1. Results and Discussion for Corrosion Mass Loss

**Figure 7:**
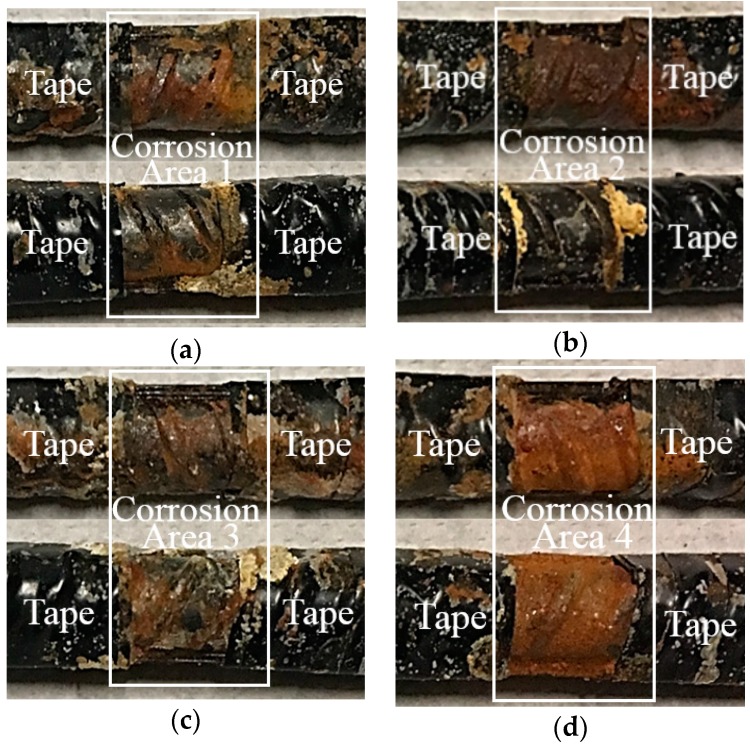
The photo assessment of the four corrosion locations with different corrosion rates. (**a**), (**b**), (**c**), (**d**) Represents the corrosion at area 1, 2, 3, 4 respectively.

The experiments were kept in a temperature-controlled environment (temperature 25 °C) to prevent a cross-sensitivity issue between strain and temperature. According to the electrochemical principle on steel rebar corrosion, the corrosion rate corresponds to the accelerated corrosion testing time. Corrosion section (1) exhibited the lowest corrosion rate and (4) exhibited the highest corrosion rate. As in Figure 7, the corrosion degree can be recognized via the surface profile of rebar. The rust products as the mass loss were measured and are listed in Table 3.

At the end of the ACT, the electrical insulation tape was removed from the steel rebar. The mass of the corroded steel rebar (*M*_0_) was measured and recorded using an electronic scale with an accuracy of 0.01 g. Acetone was used to remove the rust in section 1 of the steel rebar according to the chemical cleaning procedure described in ASTM G1-03.19 [24]. After cleaning section 1, the mass of the steel rebar was measured again (*M*_1_). This procedure was repeated until all the corroded steel rebar sections were cleaned. The total mass the steel rebar before rust removal (*M*_0_) was 326.67 g while the total mass of the steel rebar before corrosion (*M_b_*) was 326.55 g. Table 3 shows the mass measurement data, and Equation (2) was used to calculate the mass loss for each corroded steel rebar section.

(2) $$\Delta m_{i}=\left(M_{i-1}-M_{i}\right)\left[\frac{\sum_{i=1}^{n}\left(M_{i}-M_{i+1}\right)-\Delta m_{g}}{\sum_{i=1}^{n}\left(M_{i}-M_{i+1}\right)}\right]$$

where $\Delta m_{i}$ = mass loss at steel rebar section *i* (g), $M_{i}$ = total mass of steel rebar after rust removal at section *i* (g), $\Delta m_{g}$ = $M_{0}-M_{b}$ (mass gain after corrosion, g), $M_{0}$ = total mass of steel rebar before rust removal (*g*), $M_{b}=$ total mass of steel rebar before corrosion (g).

### 4.2. Ultrasound Responses for Corrosion Monitoring

**Figure 8:**
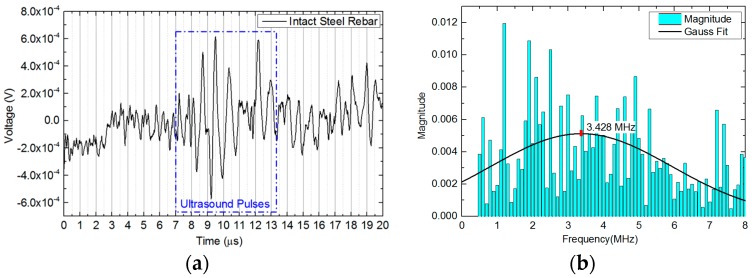
The ultrasound signals detected by FBGs on intact steel rebar. (**a**) The time domain responses of ultrasound pulses; (**b**) the frequency domain responses of ultrasound signals and Gaussian fit on frequency spectrum to obtain central frequency.

The time domain responses of ultrasound signals detected by FBGs on intact steel rebar are shown in Figure 8a. The signals were averaged 100 times. The fly time of the DAC was applied for around 20 μs. Just as theoretically calculated in Section 2.1 above, the ultrasound pulsing began at 7 μs and ended at 13 μs. At the beginning of the trigger signal, the high-density signal jump was presented as a time domain response. That was from the low-frequency noise and electromagnetic interference between the channels of trigger signal and of received signal on the DAC.

In order to analyze the spectral responses of the steel rebar, the ultrasound signals were transformed into frequency spectra via fast Fourier transformation (FFT) in Figure 8b. The central frequency in detected sections were located by using a Gaussian fit. Before the ACT processes, the bandwidth of transmitted ultrasound signals of the intact rebar was under 8 MHz and the central frequency was 3.428 MHz. The frequency spectrum of the intact rebar was selected as baseline to assess the corrosion degrees. 

### 4.3. Spectrum Responses of Corrosion Levels

**Figure 9:**
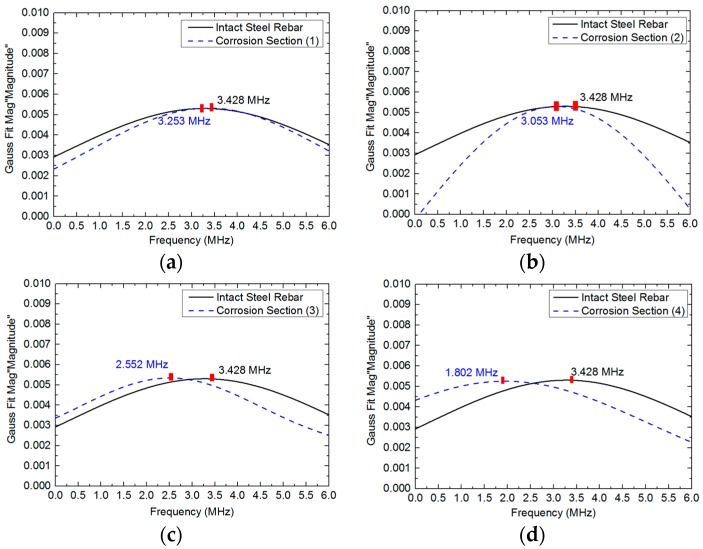
The frequency domain responses at four different corrosion sections on steel rebar after ACT.

To explore the mathematical relationship between the mass loss of the steel rebar in four different corrosion sections and the frequency spectrum responses, the ultrasound signals were detected and the central frequencies were recorded according to the spraying frequency in each section. The curves of central frequencies at four different corrosion sections are presented in Figure 9. It can be observed that the frequency components made the attenuation from high to low due to the increase of micro-corrosion products. The reduction of central frequency was 0.175 MHz at section (1), 0.375 MHz at section (2), 0.876 MHz at section (3), and 1.626 MHz at section (4), which corresponds to the corrosion degree in different sections, respectively.

**Figure 10:**
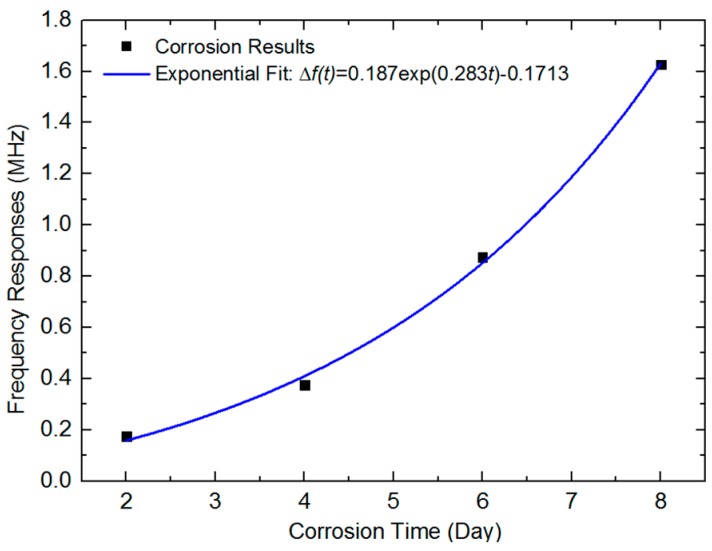
The frequency responses in four corrosion sections trendline estimates from corrosion time.

Combining the central frequency spectrum responses and corrosion time for each section, the trendline estimate is presented in Figure 10, which was fitted by using the exponential equation below:

*Δf(t)* = 0.187exp(0.283*t*) − 0.1713, (3)

where the Δf is the spectrum shift and the *t* is the corresponding corrosion time. The R-square was calculated as 0.99504 in exponential fit.

**Figure 11:**
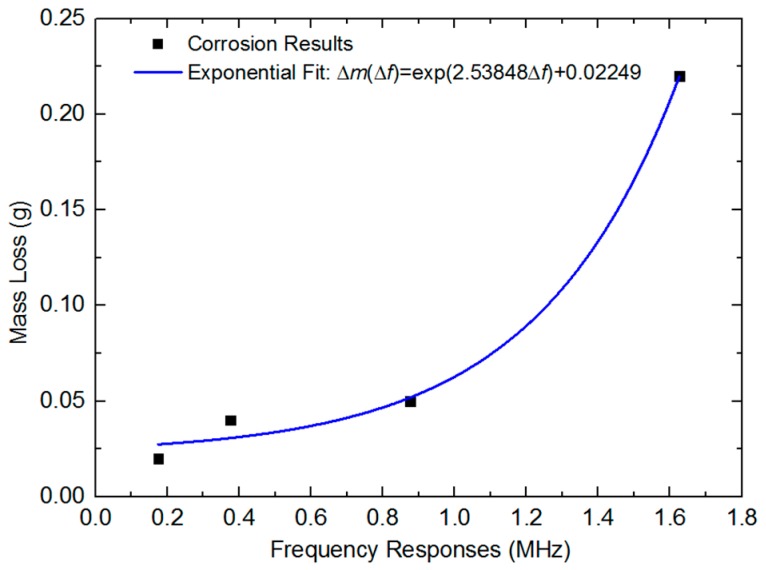
The mass loss of corrosion degree in four corrosion sections trendline estimates from frequency responses.

Combining the central frequency spectrum responses and the respective mass loss for each section, the trendline estimate is presented in Figure 11, which was fitted by using the exponential equation below:

*∆m(Δf)* = exp(2.53848 *Δf*) + 0.02249, (4)

where the Δm is the mass loss of rebar and the Δf is the corresponding spectrum shift. The R-square was calculated as 0.98306 in exponential fit. The central spectrum shifting was fitted as a perfect curve with the mass loss in exponential fit.

As shown above, the exponential equation proved to be accurate for assessing the future mass loss of steel rebar. The corrosion byproducts and microstructure changes at early stages on steel rebar were too small to specify clearly via ultrasound time domain waves. Thus, in our studies, the frequency spectra were mainly analyzed. The corrosion degree could be predicted by using the central frequency reduction in the multipoint ultrasound method. It provided a mathematical model for monitoring and specifying the corrosion level at early age.

## 5. Conclusions

In this paper, it has been demonstrated that an active distributed all-optical photoacoustic sensor could be used on steel rebar in order to detect the early signs of corrosion activity. The sensor was based on the PA principle as it applies to nanomaterials of minimal size. It converted light energy into mechanical waves with high transfer efficiency to excite ultrasound waves.

The corrosion assessment method in our studies utilized the correlation between the characteristics of pulsed ultrasound and mass loss of the steel rebar. The characteristics of these ultrasound signals were studied by extracting the signals from time domain and converting them into the frequency domain. The validity of all-optical ultrasound corrosion detection was proven by the use of accelerated corrosion testing.

The results show that the corrosion produced an exponential growth with the shifts of central frequency. The proof of such an active distributed all-optical photoacoustic sensor now leads to the development of a new approach, from passive listening to active monitoring, from one dimension node to a two-dimension surface. These findings are promising to support the efficacy of this system being embedded in concrete structures in the future.

## Figures and Tables

**Table 1:** Propagation speed of ultrasound waves in No. 4 steel rebar.

No. 4 Steel Rebar	S-Wave Velocity *C_S_*	Surface (Rayleigh) Wave Velocity *C_R_*	Propagation Time *T*
Poisson’s ratio *ν* = 0.3	*C_S_ = √(E/(p(1 + v)))*	*C_R_ = C_S_ × ((0.87 + 1.12v))/(1 + v)*	*T = L/C_R_*
Young’s Modulus *E* (GPa) = 20			
Density *p* (kg/m^3^) = 7850	3207.6 m/s	2975.7 m/s	6.9 µs

**Table 2:** Accelerated corrosion test scheme on the steel rebar.

Spraying Frequency: √ (24 h)	(1)	(2)	(3)	(4)
1	**√**	**√**	**√**	**√**
2	**√**	**√**	**√**	**√**
3	-	**√**	**√**	**√**
4	-	**√**	**√**	**√**
5	-	-	**√**	**√**
6	-	-	**√**	**√**
7	-	-	-	**√**
8	-	-	-	**√**

**Table 3:** The mass loss in four different corrosion sections.

Corrosion Section	Corrosion Time, *t* (day)	Total Mass after Rust Removal, *M_i_* (g)	Mass Loss, *Δm* (g)
(1)	2	326.64	0.02
(2)	4	326.58	0.04
(3)	6	326.51	0.05
(4)	8	326.21	0.22

## References

[1] Zhang H., Liao L., Zhao R., Zhou J., Yang M., Xia R. (2016). The non-destructive test of steel corrosion in reinforced concrete bridges using a micro-magnetic sensor. Sensors.

[2] Bastidas-Arteaga E., Stewart M.G. (2016). Economic assessment of climate adaptation strategies for existing reinforced concrete structures subjected to chloride-induced corrosion. Struct. Infrastruct. Eng..

[3] Sansalone M.J. (1997). Impact-Echo: The complete story. ACI Struct. J..

[4] Kundu T. (2012). Ultrasonic and Electromagnetic NDE for Structure and Material Characterization: Engineering and Biomedical Applications.

[5] Yu T., Owusu Twumasi J., Le V., Tang Q., D’Amico N. (2017). Surface and Subsurface Remote Sensing of Concrete Structures using Synthetic Aperture Radar Imaging. J. Struct. Eng..

[6] Bremer K., Weigand F., Zheng Y., Alwis L.S., Helbig R., Roth B. (2017). Structural Health Monitoring Using Textile Reinforcement Structures with Integrated Optical Fiber Sensors. Sensors.

[7] Barrias A., Casas J.R., Villalba S. (2016). A review of distributed optical fiber sensors for civil engineering applications. Sensors.

[8] Zhao X., Cui Y., Wei H., Kong X., Zhang P., Sun C. (2013). Research on corrosion detection for steel reinforced concrete structures using the fiber optical white light interferometer sensing technique. Smart Mater. Struct..

[9] Hu C., Yu Z., Wang A. (2016). An all fiber-optic multi-parameter structure health monitoring system. Opt. Express.

[10] Xiong W., Cai C.S. (2012). Development of Fiber Optic Acoustic Emission Sensors for Applications in Civil Infrastructures. Adv. Struct. Eng..

[11] Afzal M.H.B., Kabir S., Sidek O. Fiber optic sensor-based concrete structural health monitoring. Proceedings of the 2011 IEEE Electronics, Communications and Photonics Conference.

[12] Biagi E., Brenci M., Fontani S., Masotti L., Pieraccini M. (1997). Photoacoustic generation: Optical fiber ultrasonic sources for non destructive evaluation and clinical diagnosis. Opt. Rev..

[13] Wu N., Zou X., Zhou J., Wang X. (2016). Fiber optic ultrasound transmitters and their applications. Measurement.

[14] Fomitchov P.A., Kromine A.K., Krishnaswamy S. (2002). Photoacoustic probes for nondestructive testing and biomedical applications. Appl. Opt..

[15] Zou X., Schmitt T., Perloff D., Wu N., Yu T.Y., Wang X. (2015). Nondestructive corrosion detection using fiber optic photoacoustic ultrasound generator. Measurement.

[16] Zou X., Wu N., Tian Y., Wang X. (2014). Broadband miniature fiber optic ultrasound generator. Opt. Express.

[17] Du C., Twumasi J.O., Tang Q., Wu N., Yu T., Wang X. Real time corrosion detection of rebar using embeddable fiber optic ultrasound sensor. Proceedings of the Sensors and Smart Structures Technologies for Civil, Mechanical, and Aerospace Systems 2018.

[18] Tang Q., Du C., Wang X., Yu T. (2017). Finite Element Simulation of Real-time Health Monitoring of Steel Rod using Active Fiber Optic Sensors. Ultrasonics for Nondestructive Testing.

[19] Tang Q., Du C., Wang X., Yu T. (2016). Finite Element Simulation of a New Ultrasonic Fiber Optic Sensor using Gold Nanocomposite. Ultrasonics for Nondestructive Testing.

[20] Wu N., Tian Y., Zou X., Silva V., Chery A., Wang X. (2012). High-efficiency optical ultrasound generation using one-pot synthesized polydimethylsiloxane-gold nanoparticle nanocomposite. JOSA B.

[21] Ervin B.L., Kuchma D.A., Bernhard J.T., Reis H. (2009). Monitoring corrosion of rebar embedded in mortar using high-frequency guided ultrasonic waves. J. Eng. Mech..

[22] Li D., Zhang S., Yang W., Zhang W. (2014). Corrosion monitoring and evaluation of reinforced concrete structures utilizing the ultrasonic guided wave technique. Int. J. Distrib. Sens. Netw..

[23] Betz D.C., Thursby G., Culshaw B., Staszewski W.J. (2003). Acousto-ultrasonic sensing using fiber Bragg gratings. Smart Mater. Struct..

[24] ASTM-G1-03 (2003). Standard Practice for Preparing, Cleaning and Evaluating Corrosion Tests Specimens.

